# Ultrasound image based visual servoing for moving target ablation by high intensity focused ultrasound

**DOI:** 10.1002/rcs.1793

**Published:** 2016-12-20

**Authors:** Joonho Seo, Norihiro Koizumi, Mamoru Mitsuishi, Naohiko Sugita

**Affiliations:** ^1^ Korea Institute of Machinery and Materials Daegu South Korea; ^2^ The University of Electro Communications Tokyo Japan; ^3^ The University of Tokyo Tokyo Japan

**Keywords:** high intensity focused ultrasound, motion compensation, robotic HIFU, visual servoing

## Abstract

**Background:**

Although high intensity focused ultrasound (HIFU) is a promising technology for tumor treatment, a moving abdominal target is still a challenge in current HIFU systems. In particular, respiratory‐induced organ motion can reduce the treatment efficiency and negatively influence the treatment result. In this research, we present: (1) a methodology for integration of ultrasound (US) image based visual servoing in a HIFU system; and (2) the experimental results obtained using the developed system.

**Materials and methods:**

In the visual servoing system, target motion is monitored by biplane US imaging and tracked in real time (40 Hz) by registration with a preoperative 3D model. The distance between the target and the current HIFU focal position is calculated in every US frame and a three‐axis robot physically compensates for differences. Because simultaneous HIFU irradiation disturbs US target imaging, a sophisticated interlacing strategy was constructed.

**Results:**

In the experiments, respiratory‐induced organ motion was simulated in a water tank with a linear actuator and kidney‐shaped phantom model. Motion compensation with HIFU irradiation was applied to the moving phantom model. Based on the experimental results, visual servoing exhibited a motion compensation accuracy of 1.7 mm (RMS) on average. Moreover, the integrated system could make a spherical HIFU‐ablated lesion in the desired position of the respiratory‐moving phantom model.

**Conclusions:**

We have demonstrated the feasibility of our US image based visual servoing technique in a HIFU system for moving target treatment.

## 1 INTRODUCTION

High intensity focused ultrasound (HIFU) is well known as a promising non‐invasive tumor treatment modality, and its applications are increasing. However, treatment for moving organs is still a challenge in currently available commercial HIFU systems due to treatment efficiency and safety. Motions of abdominal organs are caused by heartbeat, respiration, or even bowel peristalsis.^1^ Any of these motions can cause a mismatch of the target position and put neighboring organs at risk during lengthy HIFU interventions. Among these organ motions, respiratory‐induced motion exhibits the largest displacement, and so substantially influences the performance of HIFU treatment.

Several tracking strategies for respiratory‐induced target motion have been introduced. In^2^ and,^3^ a 2D motion model acquired from the initial learning phase was used to correct the target position during HIFU irradiation. In MR‐guided HIFU, pencil‐beam navigator echoes were used for motion compensation of both the MR thermometry and the target position.^4^, ^5^ Auboiroux et al.^6^ combined US imaging and MR imaging for image guidance of HIFU treatment in moving tissue. Respiratory gating for HIFU ablation was also studied by,^7^, ^8^ and.^9^ In a comparison of model‐based motion compensation and respiratory gating, Rijkhorst et al.^8^ showed that the model‐based method showed lower heating rates than gating. Auboiroux et al.^7^ exploited an MR‐compatible digital camera to obtain the triggers of respiratory gating and applied the method in the liver and kidney of sheep *in vivo*. However, in respiratory gating in HIFU ablation, it is difficult to precisely deposit the heat due to the inherent heat diffusion in tissue and the reduced overall duty cycle of the gated HIFU energy delivery. Therefore, for precise ablation of a moving target, the organ motion should be monitored in real time and continuously compensated during HIFU irradiation. Diagnostic ultrasound (US) imaging is an adequate modality for real‐time monitoring of a target, is cheaper than MRI, and is easily integrated as a hardware component in HIFU systems. In,^10^ US image based visual servoing was applied to track the respiratory motion of renal stones for lithotripsy. As a visual servoing technique for soft tissue motion, in our previous research, we made an artificial marker (visible in US imaging) in the protein phantom by initial HIFU irradiation and tracked the marker for motion compensation.^11^ Chanel et al. presented a US‐guided robotic HIFU system in,^12^ which applied a speckle tracking method based on normalized 1D cross‐correlation to reduce computation time. The method is still difficult to apply for real organ motion due to the out‐of‐plane motion.

In this paper, we present a US image based visual servoing technology integrated into a HIFU system for moving target ablation. Briefly, the organ motion is monitored by biplane US imaging and the target position is calculated in real time by registration with a preoperative 3D model. The distance between the target and the current HIFU focal position becomes an error that must be compensated in the visual servoing system. In experiments, organ motion was simulated in a water tank by a linear actuator moving a kidney‐shaped phantom model. During the motion compensation by US image based visual servoing, HIFU was irradiated at the desired target position. Because simultaneous HIFU irradiation disturbs US target imaging, and particularly biplane US imaging, a sophisticated interlacing strategy of US sonication was constructed. The accuracy of the visual servoing system was evaluated by checking whether a thermal lesion was created at the desired target position and by comparing the input motion of the phantom model with the compensated motion of the end‐effector.

## 2 MATERIALS AND METHODS

### 2.1 US image based target tracking

**Figure 1:**
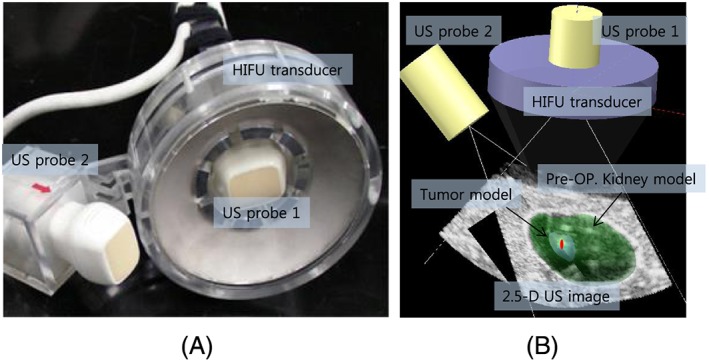
(A) End‐effector for biplane US imaging and HIFU, (B) result of preoperative 3D model registration with biplane US image model

In our HIFU system, the end‐effector enables both US imaging and HIFU irradiation by the design shown in Figure 1. The design of the end‐effector is the same as that presented in our previous research.^11^, ^13^ Two US imaging probes are attached to produce biplane US images. The US probes are connected to an ultrasonic scanner (EUB‐8500, Hitachi Medical, Inc., Japan) that runs the two sector‐type probes in turn at an imaging speed of 20 frames per second. A control PC is connected to the US diagnostic machine with a frame grabber (Matrox Meteor‐Digital, Matrox Electronic System Ltd., Canada) to capture the US raw data (IQ Data). The US data are then converted into a biplane B‐mode image with consideration of the geometry of the two US probes in the end‐effector, shown in Figure 1 To track the target position, we register the biplane US image with a preoperative 3D organ model. The 3D model based target tracking method is fast (40 FPS in the developed system) and robust for noisy US image data. The details of the registration algorithm are explained in a previously published paper.^13^

### 2.2 Visual servoing

**Figure 2:**
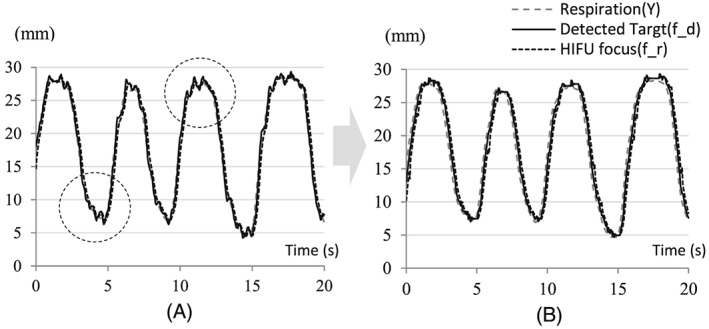
Overshoot reduction by applying encoder feedback: (A) without encoder feedback; (B) with encoder feedback

Position‐based visual servoing is implemented in a three‐axis robot system. The target position in the US image is obtained from the preoperative 3D model registration. The difference between the target position and the current HIFU focal position is considered as an error in the feedback control scheme. The control loop includes the encoder feedback (*Z*^−Δt^) to compensate for the end‐effector’’s motion during the calculation time for the current frame. The precise delay time (Δt) is measured and applied in the calculation of the new error ( $\overset{⌢}{e}$ ) as $\overset{⌢}{e}=e+Z^{-\mathrm{\Delta t}}$. When the current position is *y*, the desired target position to be compensated is *f*_*d*_, which is calculated as $f_{d}=y+\overset{⌢}{e}$. This encoder feedback plays an important role in preventing overshoot in a reciprocating motion such as respiratory motion. The experimental results in Figure 2 show the differences with and without the encoder feedback. The PID gains are fixed in both cases.

### 2.3 Integration with HIFU irradiation

**Figure 3:**
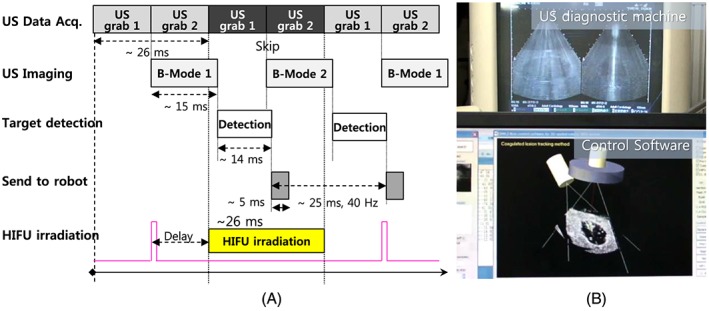
(A) Time schedule for simultaneous HIFU irradiation with real‐time tracking. (B) Image of an experimental result of interlacing US imaging and HIFU irradiation

The HIFU transducer in the end‐effector is a 100‐mm‐diameter spherical disk with a central hole of 50 mm in diameter and a spherical curvature of 100 mm for geometric focusing, as shown in Figure 1(A). Its center frequency is 1.66 MHz and the transducer is made of a single‐element piezoelectric crystal C‐213 (Fuji Ceramics, Inc., Tokyo, Japan). Since the scattered HIFU signals cause serious noise artifacts in US imaging, we need to construct a sophisticated timeline schedule in order to interlace two US sources. In particular, we need to consider the target tracking speed to secure the maximum efficiency of HIFU irradiation. A 50% duty ratio of HIFU irradiation was selected as the best setting. Therefore, we skip biplane US imaging alternately and irradiate HIFU during that time. Figure 3(A) indicates the time for each process. Based on the time schedule, the target was calculated every 25 ms, and 40 Hz target tracking was implemented. The image in Figure 3(B) is an example of interlacing based on the time schedule shown in Figure 3(A). The upper part of the image shows the noise artifacts due to HIFU irradiation in a diagnostic US machine while the lower part shows the control software displaying a B‐mode image without HIFU induced noise artifacts.

### 2.4 Phantom model for experiment

**Figure 4:**
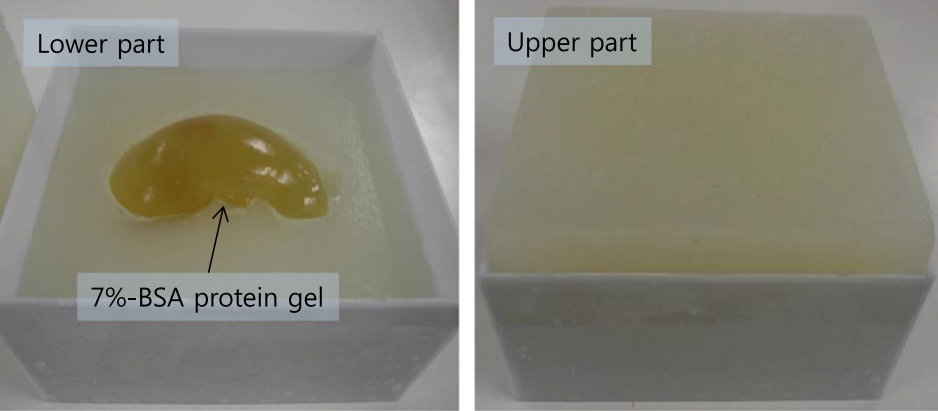
Kidney‐shaped BSA phantom model for US imaging and HIFU ablation

For the experiments of US image based visual servoing with HIFU ablation we need a phantom model that enables US imaging as well as lesion formation by HIFU thermal ablation. In this research, we developed a new phantom model combining a rectangular tissue‐mimicking phantom and a kidney‐shaped protein gel made of 7% bovine serum albumin (BSA).^14^ The protein gel was formed as the preoperative 3D kidney model and sandwiched between the tissue phantoms, as shown in Figure 4. The surface between the protein gel and the tissue phantom is shown as echogenic boundaries in the US image (see Figure 1B and Figure 3B. Therefore, the boundaries are utilized for registration with the preoperative 3D kidney model so that the target position in the US image can be calculated. Moreover, because BSA gel is optically transparent, the coagulated lesion is visually identified after HIFU ablation.

## 3 EXPERIMENTS AND RESULTS

### 3.1 Experimental conditions

**Figure 5:**
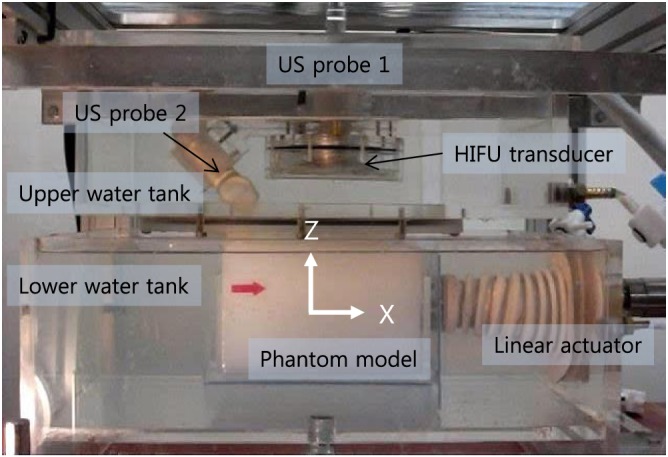
Experimental setup for biplane US image based visual servoing for HIFU irradiation

The experimental system is composed of lower and upper water tanks as shown in Figure 5.

**Figure 6:**
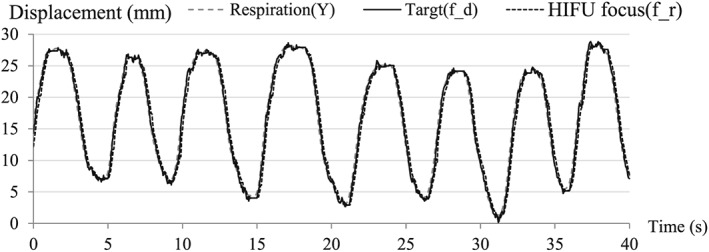
Experimental result of motion compensation with HIFU irradiation (sampling time: 40 s)

The lower water tank simulates a human body. The phantom model is submerged in the lower water tank at a water temperature maintained at 37°C. The linear actuator simulates the respiratory‐induced cranio‐caudal motion by holding the kidney phantom model. For a realistic simulation, the input motion data were captured from a real human subject: we manually aligned the US imaging plane to the cranio‐caudal axis and captured the 2D US sequence of the kidney of a healthy volunteer under free breathing. Block matching was applied to extract the displacement profile from the first frame of the 2D US sequence. Respiration (Y) in Figure 6 gives the recorded motion profile for 40 s. The maximum speed of the motion was 20 mm s^−1^ and the maximum amplitude was 25 mm.

The upper water tank has the end‐effector and contacts the surface of the phantom model via an acoustically transparent silicon membrane. The water in both tanks is degassed and circulated during the experiments.

### 3.2 Visual servoing and error analysis

To evaluate the performance of visual servoing, we recorded the detected target position (*f*_*d*_), and the position of the HIFU focus (*f*_*r*_), and the motions of the linear actuator (*Y*) simultaneously. Their errors are defined as follows:

$E_{Y‐f_{d}}$(Target detection error): error between the respiratory motion and the detected target position;
$E_{fd‐f_{r}}$(Visual feedback error): error between the detected target and the current HIFU focus;
$E_{Y‐f_{r}}$(Motion compensation error): error between the respiratory motion and the HIFU focus.

**Figure 7:**
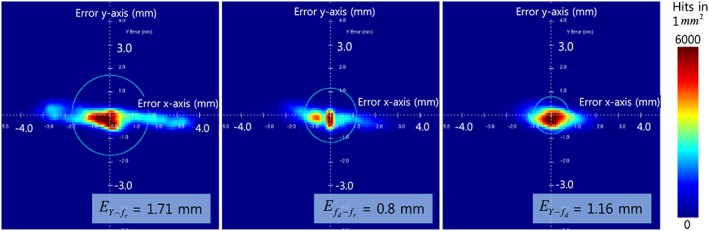
2D error histograms of motion compensation with HIFU irradiation (experimental time: 5 min): results of Experiment 3 in Table 1

As an experimental result, the time‐variant visual servoing errors over 40 s are shown in Figure 6. The gains were adjusted to track the target motion as closely as possible. The experiment for the motion‐compensated HIFU ablation was conducted for 5 min and motion data were sampled every 5 mins. The results for the total experimental time are represented as a 2D error histogram in Figure 7. Although the input motion of the phantom produced by the linear actuator was only along the x‐axis, the errors were 2D because the target detection method based on 3D model registration produced errors along the y‐axis as well. The errors along the z‐axis were negligible. We conducted the same experiment six times; the results are listed in Table 1. As shown by the results, the visual servoing system can compensate for the respiratory motion of the target with an error of 1.73 mm ( $E_{Y‐f_{r}}$, RMS) with HIFU irradiation.

**Table 1:** Experimental results: motion compensation errors with HIFU irradiation

Experiment No.	$E_{Y-f_{d}}^{\mathrm{RMS}}$	$E_{f_{d}-f_{r}}^{\mathrm{RMS}}$	$E_{Y-f_{r}}^{\mathrm{RMS}}$
1	0.77	1.16	1.74
2	0.86	1.15	1.78
3	0.80	1.16	1.71
4	0.77	1.17	1.72
5	0.81	1.18	1.76
6	0.76	1.13	1.69
Average	0.80	1.16	**1.73**

### 3.3 HIFU ablation for the moving target

**Figure 8:**
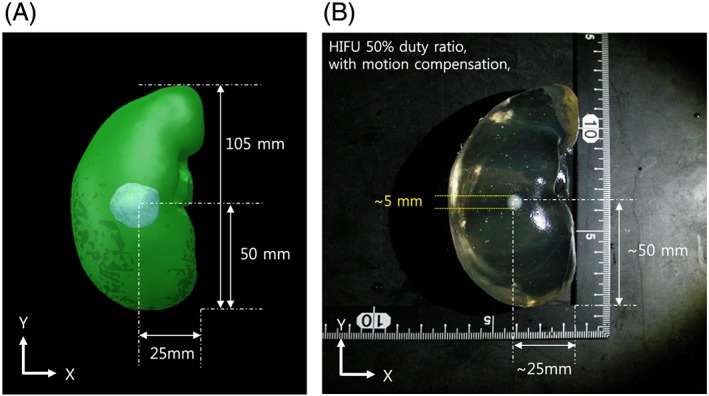
(a) Preoperative 3D kidney model with tumor volume, (b) HIFU irradiation result with motion compensation

After the visual servoing, we checked whether our HIFU system was able to create a coagulated lesion in the desired position of the moving target. The accuracy of HIFU ablation for the moving target was evaluated by comparing the position of the created lesion in the phantom model and the desired target position in the preoperative kidney model. We extracted the BSA phantom from the tissue phantom model and checked the lesion. As shown in Figure 8, the coagulated lesion was created at a position identical to that of the tumor model in the preoperative kidney phantom model. This result proves that our visual servoing was successfully applied for HIFU ablation of the moving target.

## 4 DISCUSSION

### 4.1 Motion compensation errors with HIFU thermal ablation

Because the motion compensation error is directly related to the thermal ablation efficiency of HIFU, we need to consider the relationships among motion compensation errors, HIFU irradiation time, and the size of the unit ablation area. A larger motion compensation error requires longer HIFU irradiation time to generate thermal ablation, which will make the unit ablation area larger. In the developed visual servoing system, the motion compensation error $E_{Y‐f_{r}}$ is a comprehensive error of $E_{Y‐f_{d}}$ and $E_{f_{d}-f_{r}}$. To improve $E_{Y‐f_{r}}$, therefore, we need to reduce $E_{Y‐f_{d}}$and $E_{f_{d}‐f_{r}}$. $E_{Y‐f_{d}}$, which depends on the speed of the target tracking, is reduced by adjusting the field of view of the US imaging or by reducing the calculation time. $E_{f_{d}‐f_{r}}$ depends on the hardware performance of the robot system. Increasing the control gain will decrease this error. However, the higher values of PID gain will cause a mechanical oscillation, which can increase both $E_{Y‐f_{d}}$ and $E_{f_{d}‐f_{r}}$. Without mechanical servoing, the electronic HIFU beam steering will reduce this error. However, the tracking range that the system can cover will be smaller. Because these errors in visual servoing for the HIFU system are mutually dependent, we need to carefully consider their relationships when changing any feature of a system.

### 4.2 HIFU duty ratio in the US‐guided visual servoing system

In the experimental system, US imaging and HIFU irradiation were interlaced, because the scattered HIFU signals can be a significant source of noise in US target imaging. For the interlacing, the maximum duty ratio of HIFU was set to 50% to secure the maximum US imaging speed. A higher HIFU duty ratio reduces the US imaging speed and decreases tracking accuracy, which lowers the efficiency of HIFU treatment. Therefore, 50% of the HIFU duty ratio is the optimal for current US‐guided HIFU systems. However,^15^ and^16^ proposed a method to achieve a 100% HIFU duty ratio with US imaging. The basic idea of^15^ is that the US imaging signal is encoded as a 13‐bit Barker code and is simultaneously burst with the HIFU signal. The reflected HIFU signal by the imaging array can be removed by notch filtering and pulse compression. Based on that method,^16^ introduced an adaptive method to determine the parameters of notch filtering, and the reported results seem to demonstrate an improvement over the previous method.

### 4.3 Visual servoing induced HIFU lesion control

**Figure 9:**
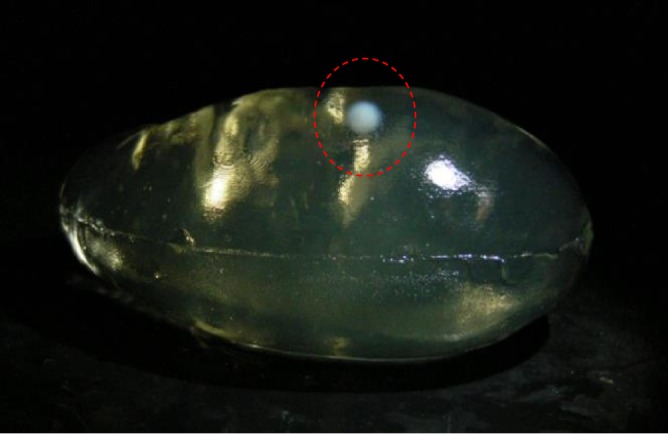
Example result of HIFU ablation with US image based visual servoing (XZ‐plane view)

No current visual servoing technique can precisely compensate for the motion of a target. Therefore, in visual servoing in the HIFU system, the error always accumulates near the target location; i.e. the heat is deposited across a region, not at a sharp point. This can be visualized by the 2D error histogram shown in Figure 7. Here, we found that the shape of the ablated lesion after motion compensation was usually spherical, as shown in the Figure 9, whereas normally the focal region is a long cigar shape^17^ along the axis of HIFU irradiation. The larger error or longer HIFU irradiation enlarges the lesion along the direction of the target motion rather than the direction of irradiation. However, appropriate conditions could be applied intentionally to create a spherical lesion. This irradiation shape might be very useful for spherical and small targets that conventional HIFU cannot treat.

### 4.4 Future improvements to US‐guided robotic HIFU systems

In the developed system, the end‐effector is submerged in a water tank and transmits US energy to the patient via a silicon membrane covering a bottom hole in the water tank. In clinical applications, therefore the water tank will be fixed on the patient's body and the end‐effector will track the target motion in the water tank. However this design limits the range of imaging and treatment, so we are planning to redesign the end‐effector with a small water bag in the next version. The FUTURA system^18^, ^19^ utilizes two robotic arms, one for the US imaging probe and the other for holding the HIFU transducer. The system gives high flexibility and enables treatment of various targets. For both cases, however, a strategy for constant skin contact should be considered. Bell et al.^20^ discussed using force control to maintain the probe contact with the patient body.

As for the US image based target tracking method, we consider applying the methods tested by De Luca et al. in.^21^ In their tests, a large number of real US sequences of the livers of both healthy volunteers and tumor patients under free breathing were used, and six different liver‐tracking methods were directly compared by a quantitative evaluation of tracking accuracy. Although the methods tested by De Luca et al. were all for liver tracking, the methods are translatable to kidneys as well.

With the developed visual servoing system, we aimed to produce a single lesion in the moving target. Therefore servoing for three translational axes was acceptable even if the target spun during the motion. However, additional degrees of freedom should be considered when multiple thermal ablations are required in the volumetric target.

## 5 CONCLUSION

In this paper, a US image based visual servoing method for a HIFU system was presented. We assumed the condition of respiratory motion, and the target organ was a kidney. In experiments, we built a moving organ model with a linear actuator and kidney‐shaped phantom model to simulate the respiratory‐induced cranio‐caudal motion of the kidney. The motion was based on real human respiration data. The kidney phantom model was monitored by biplane US imaging and the target position was calculated in real time (40 Hz) by registration of a preoperative 3D model. The distance between the target and the current HIFU focal position becomes an error that needs to be compensated in the visual servoing system. Because the motion compensation is conducted during HIFU irradiation, the system must avoid the intervention of two US sources. Therefore, a sophisticated timeline strategy was applied for US signal interlacing. Six experiments of motion compensation with HIFU irradiation were conducted, and the motion compensation error was 1.73 mm (RMS) on average. As a result, thermal lesions were created at the desired position in the moving phantom by the proposed visual servoing technique. Based on the results, we have demonstrated the feasibility of our US image based visual servoing technique in a HIFU system for moving target treatment.
